# Cyclic AMP-Dependent Protein Kinase A Regulates the Alternative Splicing of CaMKIIδ

**DOI:** 10.1371/journal.pone.0025745

**Published:** 2011-11-22

**Authors:** Qingqing Gu, Nana Jin, Hongzhuan Sheng, Xiaomin Yin, Jianhua Zhu

**Affiliations:** 1 Department of Cardiology, The Affiliated Hospital of Nantong University, Nantong, Jiangsu, People's Republic of China; 2 Jiangsu Key Laboratory of Neuroregeneration, Nantong University, Nantong, Jiangsu, People's Republic of China; New York State Institute for Basic Research, United States of America

## Abstract

Ca^2+^/calmodulin-dependent protein kinase (CaMK) IIδ is predominantly expressed in the heart. There are three isoforms of CaMKIIδ resulting from the alternative splicing of exons 14, 15, and 16 of its pre-mRNA, which is regulated by the splicing factor SF2/ASF. Inclusion of exons 15 and 16 or of exon 14 generates δA or δB isoform. The exclusion of all three exons gives rise to δC isoform, which is selectively increased in pressure-overload-induced hypertrophy. Overexpression of either δB or δC induces hypertrophy and heart failure, suggesting their specific role in the pathogenesis of hypertrophy and heart failure. It is well known that the β-adrenergic-cyclic AMP-dependent protein kinase A (PKA) pathway is implicated in heart failure. To determine the role of PKA in the alternative splicing of CaMKIIδ, we constructed mini-CaMKIIδ genes and used these genes to investigate the regulation of the alternative splicing of CaMKIIδ by PKA in cultured cells. We found that PKA promoted the exclusion of exons 14, 15, and 16 of CaMKIIδ, resulting in an increase in δC isoform. PKA interacted with and phosphorylated SF2/ASF, and enhanced SF2/ASF's activity to promote the exclusion of exons 14, 15, and 16 of CaMKIIδ, leading to a further increase in the expression of δC isoform. These findings suggest that abnormality in β-adrenergic-PKA signaling may contribute to cardiomyopathy and heart failure through dysregulation in the alternative splicing of CaMKIIδ exons 14, 15, and 16 and up-regulation of CaMKIIδC.

## Introduction

Altered intracellular Ca^2+^ handling plays an important role in the pathogenesis of cardiac hypertrophy and heart failure. Ca^2+^/calmodulin–dependent kinase II (CaMKII) is a critical transducer of Ca^2+^ signaling in the heart. Cardiac-specific overexpression of CaMKII induces a hypertrophic phenotype that rapidly transitions to dilated cardiomyopathy with ventricular dysfunction, loss of intracellular Ca^2+^ homeostasis, and premature death [1], [2], [3], [4]. Inhibition of CaMKII by either pharmacological or genetic approaches reverses heart failure–associated changes (i.e., arrhythmias, hypertrophy, and dysfunction) in animal models of structural heart disease [5], [6]. Upregulation of CaMKII expression and activity have been reported to be a general feature of heart failure in humans and in animal models [7], [8], [9], [10], [11].

**Figure 1:**
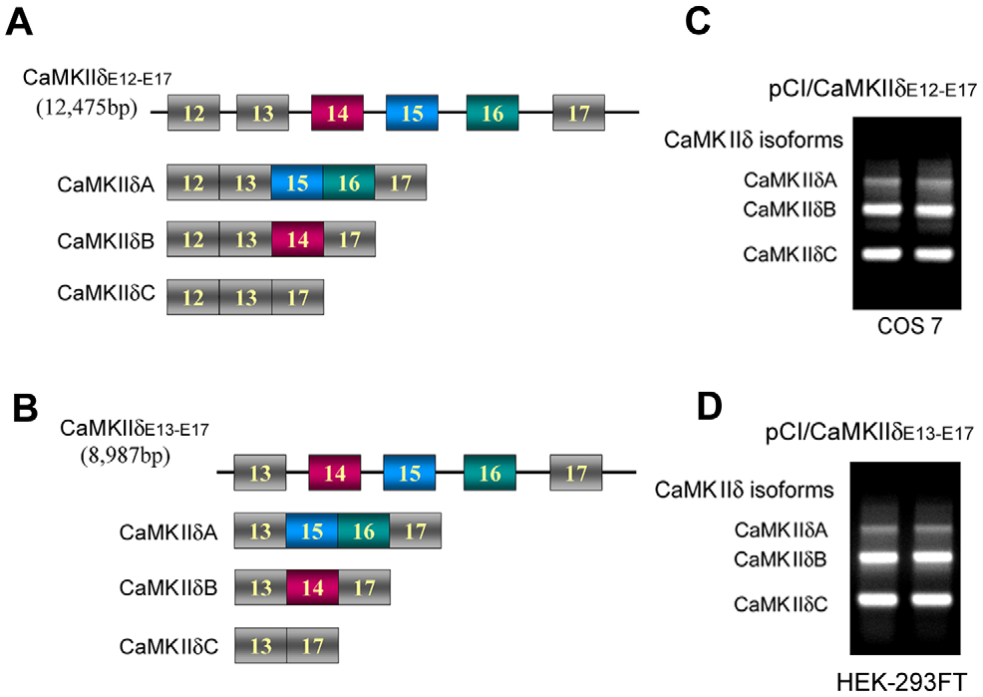
Alternative splicing of CaMKIIδ exons 14, 15, and 16 generates three splicing variants, corresponding to CaMKIIδ isoforms A, B, and C, respectively. A and B, Schematic diagram of the alternative splicing of exons 14, 15, and 16 of mini -CaMKIIδ-genes, pCI/CaMKIIδ_E12–E17_ (A) and pCI/CaMKIIδ_E13–E17_ (B). C and D, Three splicing variants was generated from mini-CaMKIIδ gene, pCI/CaMKIIδ_E12–E17_ (C) or pCI/CaMKIIδ_E13–E17_ (D), after transfection into HEK-293T or COS7 cells, respectively, for 48 hrs. The total RNA was used for measurement of the splicing products with RT-PCR.

CaMKII has four isoforms named α, β, γ andδ. CaMKIIδ is the predominant isoform in the heart [12] and is required for pathological cardiac hypertrophy and remodeling after pressure overload. Cardiomyocyte expresses three splice variants, A, B and C, of CaMKIIδ as a result of the alternative splicing of exons 14, 15 or 16 of its pre-mRNA. Inclusion of exon 15 and 16 or exon 14 generates CaMKIIδA or CaMKIIδB. CaMKIIδC is produced by exclusion of all these exons (Fig. 1A,B). The δA isoform was previously described as a neuronal CaMKIIδ isoform [13] and is associated with the T-tubules. This isoform also is expressed in neonatal heart and begins to switch off 30 days after birth [14]. The B isoform targets CaMKIIδ to nucleus due to exon 14, which contains a nuclear localization signal, and plays a key role in hypertrophic gene expression [15]. The δC isoform is the cytosolic CaMKII and affects excitation-contraction (EC) coupling through phosphorylation of Ca^2+^-regulatory proteins [16]. Overexpression CaMKIIδA in transgenic mice enhances EC coupling and induces heart failure. Moreover, transgenic mice with overexpression of either CaMKIIδB or CaMKIIδC also develop cardiac hypertrophy or heart failure [17]. Therefore, dysregulation in CaMKIIδ, including its alternative splicing, may be involved in the pathogenesis of cardiac hypertrophy and heart failure.

Splicing factor 2 or alternative splicing factor (SF2/ASF), also termed serine/arginine-rich splicing factor 1 (SRSF1), regulates both alternative splicing and constitutive splicing of many genes. Cardiac-specific-knockout of SF2/ASF causes the retention of CaMKIIδA in the adult mouse and suppression of δB and δC isoforms, suggesting that it plays critical role in the alternative splicing of CaMKIIδ [18]. SF2/ASF is a phosphoprotein. Its function and localization is highly regulated by phosphorylation. It is well known that many kinases phosphorylate SF2/ASF and regulate its biological function. We recently found that PKA phosphorylates SF2/ASF *in vitro* and in cultured cells and regulates its function in tau exon 10 inclusion [19].

β-adrenergic receptor plays a central role in sympathetic regulation of cardiac function. Catecholamines acts on β-adrenergic receptor and activates adenylyl cyclase, which catalyzes cAMP formation, via the stimulatory G protein (Gs). Subsequently, cAMP binds onto the regulatory subunits of PKA (cyclic AMP-dependent protein kinase), resulting in their dissociation from the catalytic subunits and in activation of PKA [20]. Activated PKA phosphorylates regulatory proteins involved in cardiac EC coupling and energy metabolism. It is well known that abnormalities of β-adrenergic-PKA pathway have been implicated as important determinants of cardiac hypertrophy and heart failure. Chronic heart failure is associated with an increase in circulating catecholamines [21]. Overexpression of β1-AR or Gás in transgenic mice develops cardiomyopathy and heart failure [22], [23]. The transgenic mice that express the catalytic subunit of PKA in the heart develop dilated cardiomyopathy with reduced cardiac contractility, arrhythmias, and susceptibility to sudden death. As seen in human heart failure, these abnormalities correlate with PKA-mediated hyperphosphorylation of the cardiac ryanodine receptor/Ca^2+^-release channel, which enhances Ca^2+^ release from the sarcoplasmic reticulum, and phospholamban, which regulates the sarcoplasmic reticulum Ca^2+^-ATPase [24]. Therefore, dysregulated PKA plays a specific role for in the pathogenesis of heart failure.

However, the relationship between PKA signaling and CaMKIIδ alternative splicing, both of which are related with hypertrophy and heart failure, is unclear. In the present study, by using mini-*CaMKIIδ-*gene, consisting of exons 12, 13, 14, 15, 16, and 17 and introns 12, 13, 14, 15, 16 and 17, we determined the role of PKA on the alternative splicing of CaMKIIδ. We found that SF2/ASF promoted the exclusion of exons 14, 15 and 16 and led to an increase of CaMKIIδC expression and a decrease of the expression of CaMKIIδA and δB. Phosphorylation of SF2/ASF by PKA further enhanced SF2/ASF function in the regulation of the alternative splicing of CaMKIIδ.

## Results

### Alternative splicing of exons 14, 15 and 16 generates three splicing variants of the mini-*CaMKIIδ* genes

In order to investigate the regulation of the alternative splicing of CaMKIIδ in cultured cells, we first constructed a mouse mini-CaMKIIδ-gene, pCI/CaMKIIδ_E12–E17_, consisting of exons 12,13,14,15,16 and 17, and introns 12, 13, 14, 15, and 16 (Fig. 1A), and then transfected the mini-gene into HEK-293FT cells. After 48 hr transfection, RNA was extracted and subjected to RT-PCR for measurement of the alternative splicing of exons 14, 15 and 16 of CaMKIIδ. We observed three alternative splicing products (Fig. 1C). The size of the upper band matched the product of exon 14 exclusion. The middle band was the product of exclusion of exons 15 and 16, and the lower band was the PCR product of exclusion of exons 14, 15 and 16. To confirm these three bands to be the splicing products of the mini-gene of CaMKIIδ, we sequenced the PCR products. The data showed that these three RT-PCR products were lack of exon 14, of exons 15 and 16 and of exons 14, 15, and 16, respectively (Fig. 1C). These results suggest that the mini-gene of CamKIIδ, pCI/CaMKIIδE12–E17, is able to generate three alternative splicing products of CaMKIIδ exon 14, 15 and 16, which represent CaMKIIδA, B, and C, respectively.

To test whether exon 12 and intron 12 affects the alternative splicing, we also constructed pCI/CaMKIIδ_E13–E17_ mini-CaMKIIä-gene, consisting of exons 13,14,15,16 and 17, and introns 13, 14, 15, and 16 (Fig. 1C), and transfected it into COS7 cells. After 48 hr transfection, we measured its splicing products with RT-PCR. We found that, similar to pCI/CaMKIIδ_E12–E17_, pCI/CaMKIIδ_E13–E17_ also generated three splicing products, which represented CaMKIIδA, B, and C, respectively (Fig. 1D). Thus, exons 14, 15 and 16 in these two mini-genes of CaMKIIδ, pCI/CaMKIIδ_E12–E17_ and pCI/CaMKIIδ_E13–E17_, were alternatively spliced and these two mini-genes can be used for the study of the alternative splicing of CaMKIIδ.

### PKA regulates the alternative splicing of CaMKIIδ

**Figure 2:**
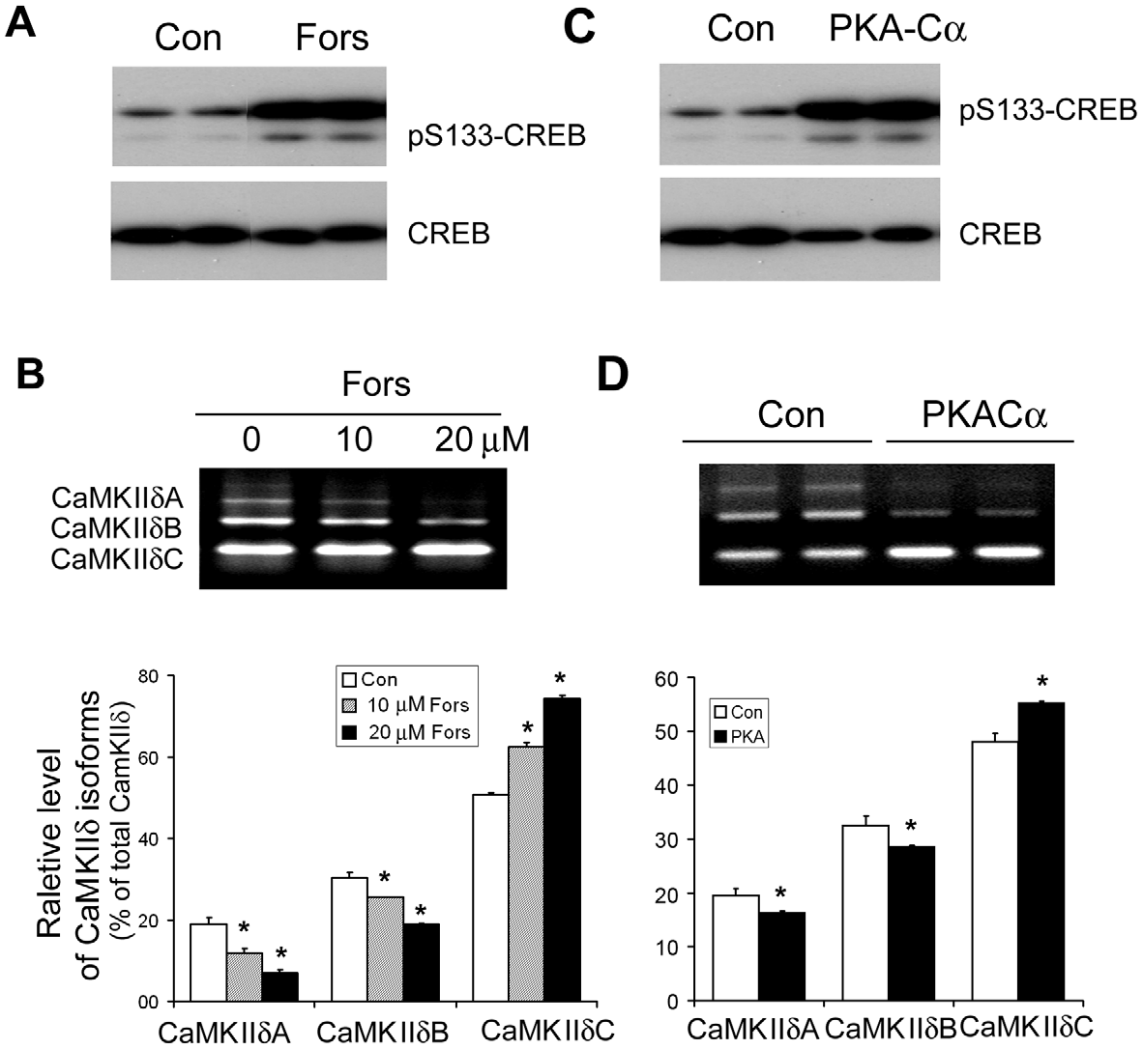
PKA promotes exclusion of exons 14, 15, and 16 of CaMKIIδ. A, Forskolin treatment activated PKA. HEK-293T cells were transfected with pCI/CaMKIIδ_E13–E17_ for 40 hrs and then treated with 10 µM forskolin for 8 hrs. The cells were subjected to Western blots for detection of PKA activity with anti-phosphorylated CREB at Ser133 and anti-CREB. B, Forskolin treatment promoted the exclusion of exons 14, 15, and 16, resulting in an increase in CaMKIIδC expression. HEK-293T cells were transfected with pCI/CaMKIIδ_E13–E17_ for 40 hrs and then treated with 10 µM forskolin for 8 hrs. The splicing products were measured with RT-PCR. Each splicing product was quantitated by densitometry, and the percentage of each splicing form was calculated. C, Overexpression of PKA-Cα increased PKA activity. HEK-293T cells were co-transfected with pCI/CaMKIIδ_E12–E17_ and pCI/PKA-Cα for 48 hrs. The PKA activity in the cells was measured by phosphorylation of CREB at Ser133 with Western blots. D, Overexpression of PKA-Cα promoted the exclusion of exons 14, 15, and 16 of CaMKII™. HEK-293T cells were cotransfected with pCI/CaMKIIδ_E13–E17_ and pCI/PKA-Cα for 48 hrs. The splicing products were measured with RT-PCR. Each splicing product was quantitated by densitometry, and the percentage of each splicing form was calculated. The Data are presented as mean ± S.D. *p<0.05 versus control treatment.

To investigate whether PKA regulates the alternative splicing of CaMKIIδ, we transfected the HEK293T cells with pCI/CaMKIIδ_E13–E17_ for 40 hr, and treated the cells with various concentration of forskolin to activate PKA for 8 hr. Forskolin treatment increased PKA activity presented by increased phosphorylation of CREB at Ser133 (Fig. 2A). We measured the alternative splicing products of CaMKIIδ exons 14, 15, and 16 with RT-PCR. We found that forskolin treatment promoted the exclusion of exons 14, 15, and 16, and suppressed the inclusion of exon 14 and of exons 15 and 16, resulting in an increase in CaMKIIδC and a decrease in CaMKIIδ A and B expression dose-dependently (Fig. 2B). These results suggest that PKA may regulate the alternative splicing of CaMKIIδ.

To confirm the regulation of forskolin on CaMKIIδ splicing via activation of PKA, we co-expressed PKA catalytic α subunit (PKA-Cα), a dominant isoform in heart [25], with the pCI/CaMKIIδ_E13–E17_, and then measured the splicing products of exons 14, 15, and 16 of CaMKIIδ. Consistent with forskolin treatment, overexpression of PKA-Cα increased PKA activity to phosphorylate CREB (Fig. 2C) and promoted the exclusion of exons 14, 15, and 16, resulting in an increase in the expression of CaMKIIδC, and a decrease in the expressions of CaMKIIδA and CaMKIIδB significantly (Fig. 2D).

### SF2/ASF promotes the inclusion of exons 14, 15, and 16 of CaMKIIδ

**Figure 3:**
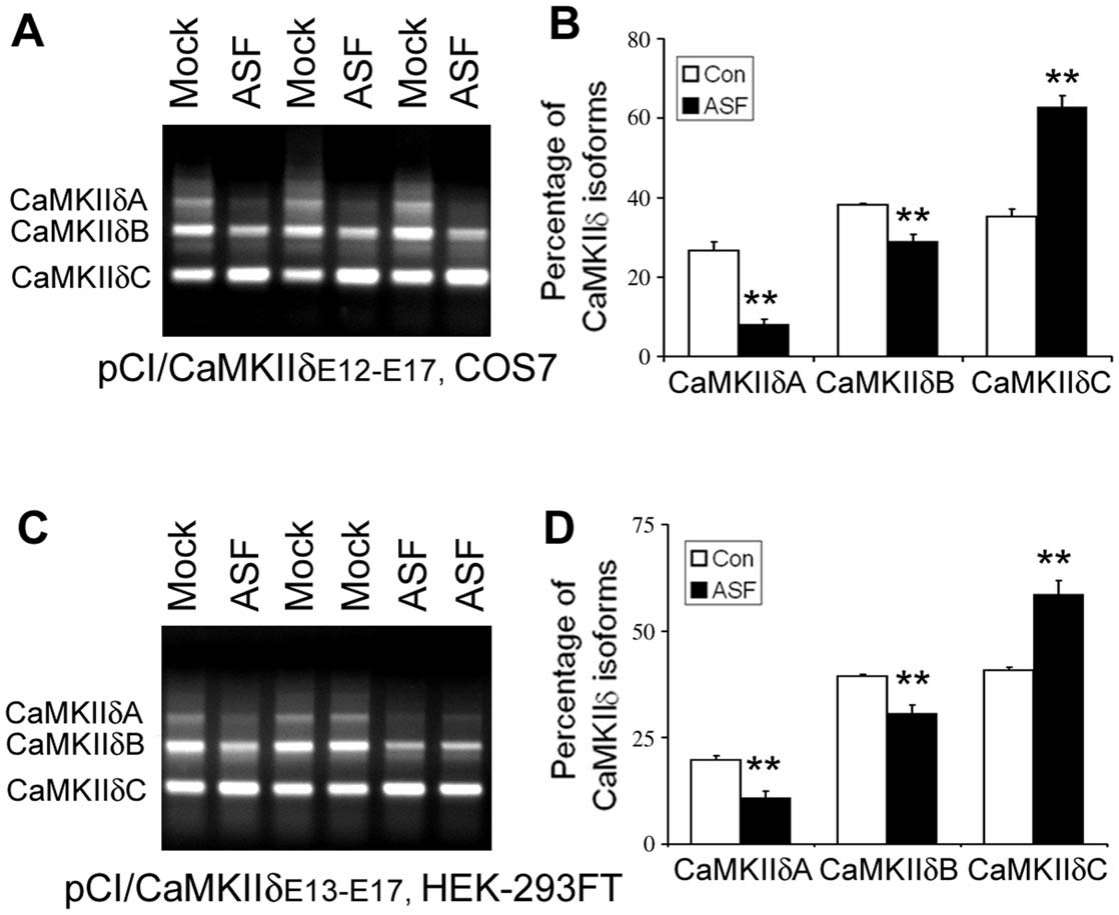
SF2/ASF promotes the exclusion of exons 14, 15, and 16 of CaMKIIδ. A and B, Overexpression of SF2/ASF promoted the exclusion of exons 14, 15, and 16 of pCI/CaMKIIδ_E12–E17_ in COS7 cells. PCI/CAMKIIδ_E12–E17_ was co-transfected with pCEP4/SF2/ASF into COS7 cells for 48 hr. The splicing products were measured with RT-PCR (A). Each splicing product was quantitated by densitometry and the percentage of each splicing form was calculated (B). C and D, Overexpression of SF2/ASF promoted the exclusion of exons 14, 15, and 16 of pCI/CaMKIIδ_E13–E17_ in HEK-239T. PCI/CAMKIIδ_E12–E17_ was co-transfected with pCEP4/SF2/ASF into HEK-293T cells for 48 hr. The splicing products were measured with RT-PCR (C). The each splicing product was quantitated by densitometry and the percentage of each splicing form was calculated (D). The Data are presented as mean ± S.D. *p<0.05 versus control treatment.

Previous studies have identified that knockout of SF2/ASF caused deregulation of the alternative splicing of CaMKIIδ [18]. To determine the regulation of SF2/ASF on the alternative splicing of exons 14, 15, and 16 of the CaMKIIδ mini-gene, we co-transfected pCEP4/SF2/ASF with pCI/CaMKIIδ_E12–E17_ into COS7 cells, and then measured the products of its alternative splicing. We found that overexpression SF2/ASF significantly promoted the exclusion of exons 14, 15 and 16, resulting in an increase of CaMKIIδC and a decrease of CaMKIIδA and B (Fig. 3A, B). Similarly, overexpression of SF2/ASF also promotes CaMKIIδC expression in pCI/CaMKIIδ_E13–E17_ transfected HEK293T cells (Fig. 3B, C). These results further confirmed that SF2/ASF could regulate the alternative splicing of exons 14, 15, and 16 and ASF promotes the exclusion of exons 14, 15, and 16 of CaMKIIδ.

### PKA phosphorylates and interacts with SF2/ASF

**Figure 4:**
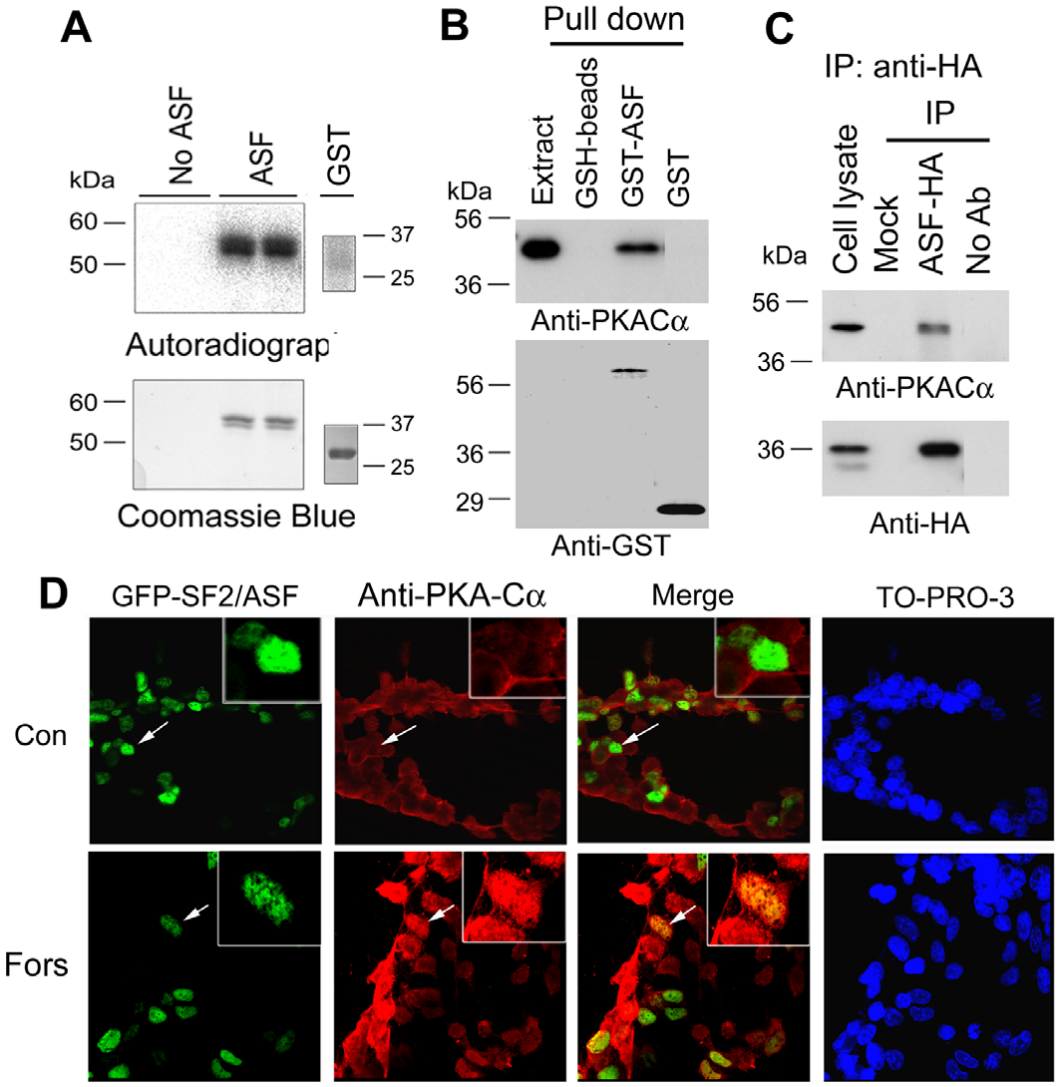
PKA phosphorylates and interacts with SF2/ASF. A, Recombinant GST-SF2/ASF or GST was incubated with PKA in the presence of [γ-^32^P]ATP at 30°C for 10 min, and the reaction mixture was then separated by SDS-PAGE and visualized with Coommassie blue staining (lower panel) or autoradiograph (upper panel). B, PKA-Cα was pull-down by SF2/ASF. GST-SF2/ASF or GST coupled onto glutathione-Sepharose or glutathione-Sepharose (GSH-beads) was incubated with rat brain extract. After extensively washing, the bound proteins were analyzed by Western blots developed with anti-GST or anti-PKA-Cα. C, PKA-Cα was co-immunoprecipitated by anti-HA. SF2/ASF tagged with HA were expressed in HEK-293FT cells for 48 h. The cell extracts were immunoprecipitated with anti-HA, and the immunoprecipitates were subjected to Western blots developed with anti-HA and anti-PKA-Cα. D, HeLa cells were transfected with pCEP4/SF2/ASF and treated without (Con) or with forskolin (Fors) for 30 min, followed by triple immunofluorescence staining.

To elucidate whether PKA regulates SF2/ASF's activity to promote the exclusion of CaMKIIδ exons 14, 15, and 16, we first determined whether PKA phosphorylates SF2/ASF in vitro. We incubated GST-SF2/ASF or GST with PKA catalytic subunit and [γ-^32^P]-ATP in reaction buffer for 30 min at 30°C, and then the reaction products were subjected to SDS-PAGE and autoradiograph for detection of ^32^P incorporation. We observed that GST-SF2/ASF, but not GST, had ^32^P incorporation, suggesting that SF2/ASF is phosphorylated by PKA (Fig. 4A).

To investigate the interaction between PKA and SF2/ASF, we performed GST pull-down assay and co-immunoprecipitation (co-IP). For GST pull down assay, we incubated GST-SF2/ASF or GST alone with rat brain extract. After extensively washing, the proteins pulled down by GST-SF2/ASF or GST were subjected to Western blots with anti-PKA-Cα. We found that PKA-Cα was pulled-down from rat brain extracts by GST-SF2/ASF, but not by GST (Fig. 4B), suggesting that SF2/ASF may interact with PKA- Cα.

Similar results were obtained from co-IP studies. We overexpressed HA tagged SF2/ASF (SF2/ASF-HA) in HEK-293FT cells and immunoprecipitated SF2/ASF with anti-HA. The immuno complex using anti-HA was subjected to Western blot analysis. We found that anti-HA immunoprecipitated SF2/ASF-HA, and that PKA-Cα subunit was co-immunoprecipitated by anti-HA. These results further suggest that SF2/ASF interacts with PKA-Cα (Fig. 4C).

To elucidate the interaction of SF2/ASF with PKA in intact cells, we transfected pCEP4/SF2/ASF into HeLa cells, and then treated the cells with 10 µM Forskolin. Their subcellular localization was determined by confocal microscopy. We observed that, without forskolin treatment, PKA-Cα was mainly located in the cytoplasm and that SF2/ASF was localized extensively in the nucleus (Data not shown). Forskolin treatment appeared to promote PKA catalytic subunit translocation into the nucleus to co-localize with SF2/ASF (Fig. 4D).

### PKA enhances SF2/ASF role in the regulation of CaMKIIδ splicing

**Figure 5:**
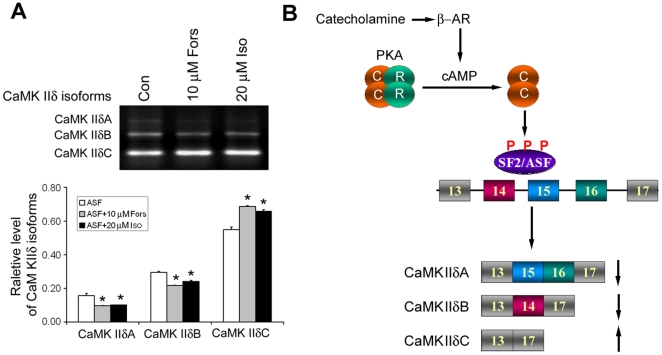
PKA activation enhances SF2/ASF-promoted exclusion of exons 14, 15, and 16 of CaMKIIδ. A, HEK-293FT cells were transfected with pCI/CaMKIIδ_E13–E17_ for 40 hrs and then treated with 10 µM forskolin or 20 µM isoproterenol for 8 hrs. The splicing products were measured with RT-PCR. Each splicing product was quantitated by densitometry and the percentage of each splicing form was calculated. The Data are presented as mean ± S.D. *p<0.05 versus control treatment. B, Proposed mechanism by which abnormalities of β-adrenergic-PKA-pathway dysregulates the alternative splicing of exons 14, 15, and 16 of CaMKIIδ via SF2/ASF.

To investigated whether PKA affects SF2/ASF-mediated alternative splicing of CaMKIIδ exons 14, 15, and 16, we co-transfected pCEP4/SF2/ASF with pCI/CaMKIIδ_E13–E17_ into HEK293T cells. After 40 hrs transfection, we treated the cells with 10 µM forskolin or 20 µM isoproterenol for 8 hr for activating PKA and then measured the splicing products of CaMKIIδ exons 14, 15, and 16. We found that both forskolin and isoproterenol treatments enhanced the role of SF2/ASF to promote the exclusion of CaMKIIδ exons 14, 15, and 16, resulting in a further increase in CaMKIIδC expression (Fig. 5).

## Discussion

The alternative splicing of exons 14, 15, and 16 of CaMKIIδ pre-mRNA generates three different splice variants, A, B and C. SF2/ASF plays an important role in the alternative splicing of CaMKIIδ [18]. In the present study, we constructed, for first time, two mini-genes of CaMKIIδ, in which exons 14, 15, and 16 were able to be alternatively spliced to generate the spliced variants corresponding to CaMKIIδA, B and C, respectively. By using these mini-genes, we confirmed the role of SF2/ASF in the promotion of exclusion of exons 14, 15, and 16 of CaMKIIδ. PKA interacted with and phosphorylated SF2/ASF and further enhanced the SF2/ASF function in promotion of exclusion of CaMKIIδ exons 14, 15, and 16. Therefore, abnormalities of β-adrenergic-PKA pathway during pathogenesis of hypertrophy and heart failure may course the dysregulation of the alternative splicing of CaMKIIδ exons 14, 15, and 16, resulting in an imbalance in the generation of CaMKIIδA, B and C isoforms, which may contribute to the hypertrophy and heart failure.

The expression of CaMKIIδ isoforms is developmentally regulated in the heart. CaMKIIδB and C are presented in the adult mammalian myocardium, but CaMKIIδA is expressed in neonatal heart and begins to switch off 30 days after birth [18]. These three isoforms of CaMKIIδ have different subcellular localization, which determines their specific roles in regulating cardiac function. The δA isoform was previously described as a neuronal CaMKIIδ isoform [13] and associates with the T-tubules where key Ca^2+^ channels and activated CaMKIIδ are concentrated [14]. As a nucleus CaMKIIδ, the B isoform plays a key role in hypertrophic gene expression [15], whiles the cytoplasmic δC isoform affects EC coupling through phosphorylation of Ca^2+^ regulatory proteins [16]. Overexpression of the δB isoform in the nucleus or the δC isoform in the cytoplasm in transgenic mice both give rise to marked decrease in diastolic Ca^2+^, transient amplitude, and caffeine-releasable Ca^2+^ content in the sarcoplasmic reticulum, all of which seem to reflect a general heart failure phenotype [4], [17].

SF2/ASF plays a crucial role in the alternative splicing of CaMKIIδ exons 14, 15, and 16. Knockout of SF2/ASF in the mouse heart promotes the inclusion of exons 15 and 16 and suppresses exclusion of exons 15 and 16 or exclusions of all exons, resulting in an increase in the expression of CaMKIIδA and a decrease in the expression of CaMKIIδB and CaMKIIδC [18]. The dysregulation of CaMKIIδ isoforms in the SF2/ASF deficient mice appears to enhance EC coupling and hypercontraction in isolated cardiomyocytes, the phenotype resulting from the targeting of the kinase to the T-tubules where several key Ca^2+^ handling proteins are located. In this study, we observed that overexpression of SF2/ASF promoted exclusion of exons 14, 15, and 16, leading to increased expression of CaMKIIδC, which is consistent with above in vivo study.

The function and distribution of SF2/ASF is highly regulated by phosphorylation. Site/regional phosphorylation impacts SF2/ASF function and subcellular localization differentially. Phosphorylation of SF2/ASF by Clk and SRSK enhances its function in the regulation of alternative splicing [26]. Phosphorylation of SF2/ASF at Ser227, Ser234 and Ser238 by Dyrk1A suppresses its function on the alternative splicing of tau exon 10 [27]. PKA phosphorylates SF2/ASF and promotes its function in the alternative splicing of CaMKIIδ. PKA is a Ser/Thr kinase and is activated by the binding of cAMP onto its regulatory subunits. We recently have demonstrated that PKA effectively phosphorylates SF2/ASF and enhances its function in promotion of tau exon 10 inclusion [19]. Here, we found that up-regulation of PKA enhances SF2/ASF-promoted exclusion of CaMKIIδ exons 14, 15, and 16, resulting in further decrease in δA and δB and increase in δC isoform of CaMKII.

Cytosolic CaMKIIδC regulates cardiac excitation-contraction coupling and maladaptive cardiac remodeling, while the nucleus form of CaMKIIδ, δB, is involved in the regulation of gene expression. Interestingly, these two major cardiac CaMKII isoforms are inversely regulated in response to oxidative stress and ischemia/reperfusion injury [28]. Overexpression of CaMKII-äC promotes apoptosis, whereas overexpression of CaMKIIδB protects cardiomyocytes against oxidative stress-, hypoxia-, and angiotensin II -induced apoptosis [28]. Overexpression of δC in the transgenic mice develops a dilated cardiomyopathy. Isolated myocytes from the mice are enlarged and exhibit reduced contractility and altered Ca^2+^ handing [4]. The expression of δC isoform of CaMKII is selectively increased and its phosphorylation elevated as early as 2 days and continuously for up to 7 days after pressure overload [4], suggesting the involvement of CaMKIIδC activation in the pathogenesis of dilated cardiomyopathy and heart failure. Down-regulation of CaMKIIδC expression may prevent hypertrophy and heart failure caused by dysregulated β-adrenergic-PKA pathway. Therefore, the roles of PKA in the regulation of alternative splicing of CamKIIδ and in the pathogenesis of cardiomyopathy and heart failure *in vivo* remain to be elucidated.

## Materials and Methods

### Plasmids, antibodies, and other reagents

CaMKIIδ minigene pCI/CaMKIIδ_E12–E17_ or pCI/CaMKIIδ_E13–E17_, comprising CaMKIIδ exons 12, 13, 14, 15, 16 and 17 and introns 12, 13, 14, 15, 16, was generated by PCR from mouse genomic DNA as described below. The expression constructs for human PKA-Cα was generated by reverse-transcription PCR from RNA isolated from normal human neuronal progenitor cells (Lonza, Walkersville, MD, USA) and confirmed by DNA sequence analysis. PKA-Cα tagged with HA was cloned into pCI vector via Sal I and Not I sites. pCEP4/SF2/ASF-HA was a gift from Dr. Tarn of the Institute of Biomedical Sciences, Academia Sinica, Taiwan. pGEX-2T/SF2/ASF was constructed by PCR amplification from pCEP4-SF2/ASF-HA and sub-cloning into pGEX-2T to express GST-fusion proteins of SF2/ASF. The catalytic subunit of PKA and the monoclonal anti-HA were bought from Sigma (St. Louis, MO, USA). Polyclonal Anti-PKA-Cα was from Abcam (Cambridge, UK) and polyclonal anti-PKA-Cβ was from Santa Cruz Biotechnology (Santa Cruz, CA, USA). Peroxidase-conjugated anti-mouse and anti-rabbit IgG were obtained from Jackson ImmunoResearch Laboratories (West Grove, PA, USA); Alexa 488-conjugated goat anti-mouse IgG, Alexa 555-conjugated goat anti-rabbit IgG and TO-PRO-3 iodide (642/661) were from Invitrogen (Invitrogen, Carlsbad, CA, USA). ECL Kit was from Amersham Pharmacia (Amersham Bioscience, Piscataway, NJ, USA), and [γ-32P]ATP and [^32^P]-orthophosphate were from MP Biomedicals (Irvine, CA, USA).

### Construction of mini-*CaMKIIδ-*genes, pCI/CaMKII*δ*_E12–E17_ and pCI/CaMKIIδ_E13–E17_

C57BL6 mouse was from the laboratory animal center of Nantong University. The protocol was approved according to the Animal Care and Use Committee of Nantong University and the Jiangsu Province Animal Care Ethics Committee (Approval ID: SYXK(SU)2007-0021). Mouse genomic DNA was extracted from C57BL6 mouse tail with Dneasy Blood and Tissue kit (Qiagen, Germany) and used as a template to amplify genomic DNA of CaMKIIδ from exon 12 to exon 17 including introns (E12–E17, 12,475 bp) with two pairs of primers (Fig. 1a). Set 1 of primers (forward, 5′cgACGCGTatttctttgatagggcgccatcttgacaac3′; reverse, 5′acgcGTCGACaaagagccttcttctgtgg3′) was used to generate 6825 bp fragment. Set 2 of primers (forward, 5′acgcGTCGACacatgggagcttggaatg3′; reverse, 5′ataagaatGCGGCCGCgcctcaagcctacctttcacgtcttcatcc3′). The PCR products were digested by *Mlu I* and *Sal I*, and *Sal I* and *Not I* respectively, and inserted into pCI/neo vector by restriction sites of *Mlu I* and *Not I*, which named pCI/CaMKIIδ_E12–E17_. The sequences of the mini-gene was confirmed by sequencing analysis.

By using pCI/CaMKIIδ_E12–E17_ as the template, we amplified the genomic DNA of CaMKIIδ from exon 13 to exon 17 with primers (forward, 5′cgACGCGTtcattttgca gcagccaagagtttattgaag3′; reverse, 5′ataagaatGCGGCCGCgcctcaagcctacctttcacgtcttcatcc3′) to amplify the genomic DNA of CaMKIIδ from exon 13 to exon 17 and inserted into pCI/neo by *Mlu I* and *Not I*, which was named pCI/CaMKIIδ_E13–E17_.

### Cell culture and transfection

COS-7, HeLa, and HEK-293FT cells (ATCC, Manassa, VA, USA) were maintained in Dulbecco's modified Eagle's medium (DMEM) supplemented with 10% fetal bovine serum (Invitrogen) at 37°C. All transfections were performed in triplicate with FuGENE 6 (Roche Diagnotics, Indianapolis, IN, USA) in 12-well plate or 4 well-chamber. The cells were transfected with FuGENE 6 for 48 h according to the manufacturer's instructions.

### *In vitro* phosphorylation of SF2/ASF by PKA

GST and GST-SF2/ASF (0.2 mg/ml) was incubated with PKA catalytic subunit in a reaction buffer consisting of 50 mM 40 mM HEPES (pH 6.8), 10 mM β-mercaptoethanol, 10 mM MgCl2, 1.0 mM EGTA and 0.2 mM [γ-^32^P]ATP (500 cpm/pmol). After incubation at 30°C for 30 min, the reaction was stopped by boiling with an equal volume of 2×Laemmli sample buffer. The reaction products were separated by SDS-PAGE. Incorporation of ^32^P was detected by exposure of the dried gel to phosphor-image system.

### GST pull-down of PKA by SF2/ASF

GST and GST-SF2/ASF were purified by affinity purification with glutathione-Sepharose, but without elution from the beads. These beads coupled with GST and GST-SF2/ASF were incubated with crude extract from rat brain homogenate in buffer (50 mM Tris-HCl, pH 7.4, 8.5% sucrose, 50 mM NaF, 1 mM Na_3_VO_4_, 0.1% Triton X-100, 2 mM EDTA, 1 mM PMSF, 10 µg/ml aprotinin, 10 µg/ml leupeptin and 10 µg/ml pepstatin). After 4 h incubation at 4°C, the beads were washed with washing buffer (50 mM Tris-HCl, pH7.4, 150 mM NaCl and 1 mM DTT) six times, the bound proteins were eluted by boiling in Laemmli sample buffer and the samples were subjected to Western blot analysis.

### Co-immunoprecipitation of PKA by SF2/ASF

HEK-293FT cells were transfected with pCEP4-SF2/ASF-HA for 40 h as described above and treated with 10 uM forskolin for 8 h, and then the cells were washed twice with PBS, and lysed by sonication in lysate buffer (50 mM Tris-HCl, pH 7.4, 150 mM NaCl, 50 mM NaF, 1 mM Na_3_VO_4_, 2 mM EDTA, 1 mM PMSF, 2 µg/ml aprotinin, 2 µg/ml leupeptin and 2 µg/ml pepstatin). The cell lysate was centrifuged at 16,000×g for 10 min and incubated with anti-HA overnight at 4°C, and then protein G beads were added. After 4 h incubation at 4°C, the beads were washed with lysate buffer twice and with TBS twice, and bound proteins were eluted by boiling in Laemmli sample buffer. The samples were subjected to Western blot analysis with the indicated primary antibodies.

### Co-localization of PKA with SF2/ASF

HeLa cells were plated in 24-well plates onto coverslips one day prior to transfection at 50–60% confluence. The cells were then transfected with pCEP4/SF2/ASF-HA as described above. After 40 h transfection, the cells were treated with 10 µM forskolin for 30 min to activate PKA, and then the cells were washed with PBS and fixed with 4% paraformaldehyde in PBS for 30 min at room temperature. After washing with PBS, the cells were blocked with 10% goat serum in 0.2% Triton X-100-PBS for 2 h at 37°C, and incubated with rabbit anti-HA antibody (1∶200) and mouse anti-PKACα (1∶50) overnight at 4°C. The cells were then washed and incubated for 1 h with secondary antibodies (Alexa 488-conjugated goat anti-mouse IgG and Alexa 555-conjugated goat anti-rabbit IgG, 1∶1000) plus TO-PRO-3 iodide at room temperature. The cells were washed with PBS, mounted with Fluoromount-G and observed with a Nikon TCS-SP2 laser-scanning confocal microscope.

### Quantitation of splicing products of CaMKIIδ exons 14, 15, and 16 by reverse transcription-PCR (RT-PCR)

Total cellular RNA was isolated from cultured cells by using the RNeasy Mini Kit (Qiagen, GmbH, Germany). One microgram of total RNA was used for first-strand cDNA synthesis with Oligo-(dT)_15_–_18_ by using the Omniscript Reverse Transcription Kit (Qiagen). PCR was performed by using PrimeSTAR™ HS DNA Polymerase (Takara Bio Inc., Otsu, Shiga, Japan) with primers (forward, 5′ GGTGTCCACTCCCAGTTCAA 3′; reverse, 5′ GTCTTCATCCTCAATGGTGG TG 3′) to measure alternative splicing of CaMKIIδ exons 14, 15, and 16. The PCR conditions were: 98°C for 3 min, 98°C for 10 sec and 68°C for 40 sec for 25–30 cycles and then 68°C 10 min for extension. The PCR products were resolved on 1.5% agarose gels. Each splicing product was quantitated by densitometry using the Molecular Imager system (Bio-Rad, Hercules, CA, USA), and the percentage of each splicing form was calculated.

### Statistical analysis

All data are expressed as the means±SD. Data points were compared by unpaired two-tailed Student's *t* test. A value of *p*<0.05 was considered statistically significant.
